# Synergy Feedback Control Predicts Walking Across Multiple Cycles

**DOI:** 10.64898/2026.03.02.709098

**Published:** 2026-03-04

**Authors:** Spencer Williams, Geng Li, B.J. Fregly

**Affiliations:** 1 Rice Computational Neuromechanics Lab, Department of Mechanical Engineering, Rice University, Houston, TX, United States

**Keywords:** Neuromusculoskeletal modeling, Predictive simulation, Model Personalization, Treatment Optimization, EMG-driven modeling, Muscle synergies, Neural feedback, Stroke

## Abstract

Neural feedback is important for healthy control of movement, and multiple neurological disorders (e.g., stroke, cerebral palsy, Parkinson’s disease, incomplete spinal cord injury) can be described by how they impair healthy feedback or induce unhealthy feedback. Researchers have created numerous computational neuromusculoskeletal models controlled by simulated neural feedback mechanisms, but these models rarely represent actual human subjects and thus have not found practical application in treating patients with movement impairments. As a step toward designing patient-specific treatments for individuals with neurological disorders, this study used the Neuromusculoskeletal Modeling Pipeline to develop and evaluate a novel synergy-based feedforward (FF)+feedback (FB) model using a personalized, three-dimensional neuromusculoskeletal walking model of an actual human subject post-stroke. Experimental walking data collected from the subject were used to create the subject’s personalized walking model. This model was used to calculate lower body muscle activations consistent with the subject’s electromyographic, joint motion, and ground reaction data for 5 calibration walking cycles. Nominal FF synergy controls were calculated by averaging the muscle synergies that closely reconstructed the 5 cycles of muscle activations and associated joint moments simultaneously. These nominal FF controls were then scaled by 0, 25, 50, 75, 100, and 125%, and the gap in reproducing individual cycle muscle activations was filled by fitting FB synergy controls as a function of joint positions, velocities, and moments as surrogates for muscle lengths, muscle velocities, and tendon forces. Finally, the six synergy-based FF+FB models controlled the subject’s personalized walking model in predictive simulations performed for 3 testing walking cycles withheld from calibration. The 100% FF model (which still had minimal FB) reproduced the testing walking cycles the most closely, and only the 75%, 100%, and 125% FF models generated near-periodic walking motions using initial conditions consistent with experimental values. The 0, 25, and 50% FF models could generate near-periodic walking motions only when the initial conditions were allowed to diverge substantially from experimental values. Our findings suggest that predictive simulations of walking using real experimental data may require a minimum level of feedforward control and sufficient fitting data to predict a subject’s actual dynamically consistent motion.

## 1 Introduction

Neural feedback is an essential component of the neuromuscular control of movement. Healthy neural feedback helps individuals adapt their walking to new situations or perturbations (Severini and Zych, 2022), and reflexes prevent dangerous slips and falls (McCrum et al., 2017). Disruptions to these mechanisms can cause movement impairments and health hazards, with conditions producing muscle spasticity, a harmful velocity-dependent feedback mechanism (Jansen et al., 2014; Trompetto et al., 2014; Ang et al., 2018; Falisse et al., 2018). Detrimental neural feedback is present in conditions such as cerebral palsy (Falisse et al., 2018), spinal cord injuries (Trompetto et al., 2014; Ang et al., 2018), stroke (Jansen et al., 2014; Ang et al., 2018), and Parkinson’s disease (Kim et al., 2009). However, a thorough understanding of how these detrimental feedback mechanisms work remains elusive, hindering the development of more effective treatments for these conditions.

Researchers have been exploring the use of computational neuromusculoskeletal models to develop a better understanding of human reflex contributions to walking and movement impairments (Geyer and Herr, 2010; Song and Geyer, 2013; Jansen et al., 2014; Falisse et al., 2018; Haeufle et al., 2018; Koseki et al., 2024). Past work shows that while complicated neural networks in the brain control movement, simple walking models can be successfully controlled by feedback mechanisms alone (Geyer and Herr, 2010). These models can be made more robust by combining feedforward and feedback control (Haeufle et al., 2018). Similar models have walked and ran at different speeds using similar control structures activating muscle synergies rather than individual muscles (Aoi et al., 2019). In an experimental study of subjects attempting to stand upright while being perturbed, muscle synergy controls could be fitted using feedback quantities related to a target motion, such as center of mass location (Safavynia and Ting, 2013), further supporting the use of muscle synergies in human movement simulations. Modeling studies have since explored many formulations of feedback control for walking (Aoi et al., 2019; Di Russo et al., 2021; Lassmann et al., 2023; Su and Gutierrez-Farewik, 2023). It is notable, however, that most past simulation studies have predicted general movement characteristics or limited experimental data rather than the response of specific individuals under new conditions. Thus, use of patient-specific neural feedback models would likely be beneficial when the goal is to develop treatments for movement impairments caused by neurological disorders.

As a step toward achieving this goal, this study describes the development and evaluation of a novel process for creating personalized feedforward (FF)+feedback (FB) neural control models to predict three-dimensional walking motions for an individual post-stroke. The study is made possible by a personalized neuromusculoskeletal walking model created from experimental walking data collected from the individual. Personalized FF+FB neural control models are implemented using muscle synergies. Models with different ratios of FF to FB control are calibrated using 5 cycles of walking data and tested in predictive simulations using 3 additional cycles of walking data withheld from calibration. Our hypothesis was that the neural control model with the least reliance on FB would have the strongest predictive ability due to its relatively low sensitivity to FB inputs, though we expected all FB models tested to be capable of producing walking-like motions. Personalized neural feedback models similar to the ones investigated here may eventually help researchers and clinicians identify optimal treatment plans for individuals with movement impairments caused by various neurological disorders.

## 2 Materials and Methods

### 2.1 Experimental Data

For this modeling study, we used walking data previously collected with informed consent from a male stroke survivor (age 79 years, LE Fugl-Meyer Motor Assessment 32/34 pts, right-sided hemiparesis, height 1.7 m, mass 80.5 kg) (Meyer et al., 2016). The data included marker-based motion capture data using a modified Cleveland Clinic full-body marker set with additional foot markers (Reinbolt et al., 2005) (Vicon Corp., Oxford, UK), ground reaction force and moment data from a split-belt instrumented treadmill (Bertec Corp., Columbus, OH, USA), and electromyographic (EMG) data from 16 muscles per leg (Motion Lab Systems, Baton Rouge, LA, USA). Data collected during a standing static pose were used for model scaling purposes (see below), while data collected during 30 seconds of treadmill walking at a self-selected speed of 0.5 m/s were used for the feedback control study.

EMG data for each leg were collected using a combination of surface and fine wire electrodes. Surface electrodes were used for gluteus maximus and medius, semimembranosus, biceps femoris long head, rectus femoris, vastus medialis and lateralis, medial gastrocnemius, tibialis anterior, peroneus longus, and soleus. Fine wire electrodes were used for adductor longus, iliopsoas, tibialis posterior, extensor digitorum longus, and flexor digitorum longus. Raw EMG data were converted to muscle excitation envelopes using standard EMG processing methods (Lloyd and Besier, 2003).

### 2.2 Neuromusculoskeletal Modeling

#### 2.2.1 Joint and Ground Contact Model Personalization

A generic full-body OpenSim (Seth et al., 2018) musculoskeletal model (Hammond et al., 2025) derived from a previously published OpenSim model (Rajagopal et al., 2016) was used as a baseline. Forearm pronation-supination, and knee adduction DOFs were locked, leaving the generic model with 31 DOFs. The DOFs included a 6 DOF ground-to-pelvis joint, two 3 DOF hip joints, two 1 DOF knee joints, two 1 DOF ankle joints, two 1 DOF subtalar joints, two 1 DOF toe joints linking the hindfoot and toes, a 3 DOF back joint, two 3 DOF shoulder joints, and two 1 DOF elbow joints. The model included 86 Hill-type muscle-tendon actuators with rigid tendons (43 per leg). The model was scaled in OpenSim using the static pose recording with bodies and joints scaled symmetrically.

The skeletal geometry and joint parameters of the scaled model were personalized to the subject’s experimental data using the Joint Model Personalization tool of the Neuromusculoskeletal Modeling (NMSM) Pipeline (Hammond et al., 2025). The tool optimized joint axis parameters, uniform body scaling, and marker locations to reduce inverse kinematics (IK) marker distance errors when attempting to reproduce the motion of a portion of the 30 second walking trial. The tool was allowed to change joint axis orientation parameters in the knee, ankle, and subtalar joints, uniform scaling of the pelvis, femur, and tibia bodies, and the locations of markers attached to the femur and tibia bodies. The cost function penalized IK marker distance errors. The calibrated model was then used with OpenSim’s Inverse Kinematics tool to solve for joint angles that reproduced the experimental marker motion data as closely as possible. The IK results were filtered using a fourth-order zero phase lag Butterworth filter with a cutoff frequency of 7/tf Hz, where tf is the average period of a gait cycle from the 30 second recording (Hug, 2011).

After the skeletal structure was personalized and IK joint angles were calculated, the Ground Contact Personalization tool within the NMSM Pipeline was used to generate subject-specific elastic foundation foot-ground contact models. Prior to running the tool (and for future steps), the experimental ground reaction forces and moments were filtered using a second-order zero phase lag Butterworth filter with a cutoff frequency of 7/tf Hz.

The tool automatically placed 37 viscoelastic contact elements across the hindfoot and toes segment of each foot and calibrated each contact element’s stiffness coefficient, a common nonlinear damping coefficient, a common dynamic friction coefficient, and a common resting spring length defining the height at which the contact elements penetrated the ground. To calibrate these parameters, the optimization cost function penalized errors in reproducing all three components of the experimental ground reaction forces and moments while making minimal adjustments to the experimental foot kinematics with respect to ground. To prevent unrealistic discontinuities in contact element stiffness values, the cost function also minimized the deviation of each contact element stiffness value away from neighboring values using a Gaussian-weighted average, where stiffness values from nearer elements had a higher weight. In addition, to make the ground reaction data more consistent with the marker motion data and the dynamics of the skeletal model, the cost function made small adjustments to the location of each force plate in the lab coordinate system (Williams et al., 2025). The adjusted ground reaction data were used when calculating joint moments with OpenSim’s Inverse Dynamics (ID) tool.

#### 2.2.2 Torque-Driven Tracking Optimization

From the full 30 seconds of walking data, 13 well-defined gait cycles starting with the right foot heelstrike were identified, and the IK, ID, and ground reaction data were split into separate data files for each gait cycle, each splined to 101 time points. The data for each gait cycle were used to initialize variables and provide tracked quantities for direct collocation optimal control problems deriving dynamically consistent torque-driven walking motions. The Tracking Optimization (TO) tool in the NMSM Pipeline was used to perform these optimizations. The values of coordinate positions, velocities, and accelerations were allowed to change, resulting in different ground reactions and joint loads, and torque controls were applied to the hip, knee, ankle, and subtalar coordinates. The cost function minimized tracking errors in joint angles, joint velocities, joint accelerations, ground reaction forces and moments, ID joint loads, and heel marker positions and velocities. Constraints were used to maintain joint coordinate and joint speed periodicity and initial and final values of ground reaction forces and moments. Additional constraints enforced that torque controls stayed within 0.1 Nm of their respective ID joint loads calculated during the optimization and that residual ID loads applied to the pelvis, corresponding to the coordinates of the 6 DOF ground-to-pelvis joint, were less than 1 N or 0.1 Nm in magnitude. Of the 13 trials used with the TO tool, the 8 trials with the lowest overall tracking errors were selected for use in future steps. Joint angle, ID, and ground reaction data from the dynamically consistent TO solutions were used for these 8 trials in place of their experimental data.

#### 2.2.3 Muscle-tendon and Neural Control Model Personalization

The Muscle Tendon Personalization (MTP) tool of the NMSM Pipeline was used to calibrate muscle-tendon model properties. For each of the 8 trials, time-varying muscle length, velocity, and moment arm data were calculated for each muscle, with moment arms about each coordinate, based on updated joint angles. These data were filtered with using the same protocol as IK joint angles. EMG data were high-pass filtered at 40 Hz, demeaned, rectified, low-pass filtered with a cut-off frequency of 3.5/tf Hz, normalized to a maximum value of 1, split into separate data files for each trial, and splined to 101 time points plus a cycle-dependent number of padding frames, adding 200 ms before and after the time ranges used by ID data to allow for electromechanical delay. The MTP tool calibrated muscle-tendon parameters and solved for time-varying activations for each muscle from EMG data. Muscle-specific electromechanical delays, activation time constants, activation nonlinearity constants, EMG scale factors, optimal fiber lengths, and tendon slack lengths were solved, as well as synergy-based estimates of unmeasured excitations for hip rotators, tensor fasciae latae, and sartorius muscles that were not adequately described by the collected EMG were optimized. The cost function penalized: errors in hip flexion, hip adduction, knee, ankle, and subtalar joint moments produced by muscles relative to ID loads from the torque-driven TO simulations; high extrapolated muscle excitations; changes to measured excitations made by the extrapolation process (Ao and Fregly, 2024); deviations from error centers in activation time constants, activation nonlinearity constants, optimal fiber lengths, tendon slack lengths, EMG scale factors, and normalized muscle fiber lengths; high passive muscle forces; high muscle excitations; deviations within defined muscle groups in normalized fiber length, EMG scale factors, and electromechanical delays. The optimization was performed on all 8 cycles simultaneously to ensure all trials used consistent muscle-tendon properties.

Next, the Neural Control Personalization (NCP) tool within the NMSM Pipeline was used to impose a synergy structure on the MTP muscle activations. From the 8 cycles, the 5 least similar in IK, ID, and ground reaction data to deviations from the mean were selected for fitting a neural feedback model to capture the most variation available during the calibration process. The remaining 3 cycles were set aside for testing feedback models. Using the 5 fitting cycles, NCP solved for 5 sets of synergy weights and commands per leg, using a highly weighted cost term to keep synergy weights almost identical between the two sides to enable direct comparison of their activation levels. The cost function penalized errors in tracking known joint moments and muscle activations and deviations within muscle groups in muscle activations. Synergy commands were produced for each cycle, and all 5 cycles shared a set of synergy weights. The means of the synergy activations for all 5 trials, calculated at each of the 101 time points, were used as feedforward synergy commands in future steps.

Finally, feedforward commands, joint angles, and ID moments were used to fit neural feedback weights that would best reproduce cycle-specific synergy commands for the 5 fitting cycles. Total synergy commands were calculated by adding each mean feedforward command to a corresponding feedback command calculated by a linear feedback model. Hip flexion and adduction, knee, ankle and subtalar joint angles, velocities and joint moments for both sides were used as feedback inputs. To test a variety of balances in magnitude between feedforward and feedback commands, feedback weights were fit with feedforward commands scaled to 0%, 25%, 50%, 75%, 100%, and 125% their nominal magnitudes. A MATLAB nonlinear least-squares optimizer solved for feedback weights for each set of scaled feedforward commands, with a cost function penalizing errors in cycle-specific synergy commands and high feedback weight magnitudes.

#### 2.2.4 Synergy-Driven Predictive Simulations with Neural Feedback

Each of the 6 combined feedforward+feedback models was used in a synergy-driven predictive simulation performed using the TO tool to evaluate how well 3 testing gait cycles could be reproduced. The cost function penalized errors in upper-body, hip rotation, and toes joint angles and velocities and torso orientation and angular velocity with respect to ground. These cost terms accounted for portions of the model that were not controlled directly by the feedback model. Feedforward commands were included in the optimal control problem as controls and constrained to have changes of less than 0.01, thereby enforcing their shapes without negatively impacting the performance of the optimization. Additional constraints were used to ensure that initial values of joint angles, joint velocities, and ground reactions remained close to experimental initial values, that the predicted motion was near-periodic, that dynamic consistency was maintained by keeping muscle-produced joint moments within 1 Nm of their corresponding ID joint loads, and that residual ID loads applied to the pelvis were less than 1 N or 0.1 Nm in magnitude. The problems using 0%, 25%, and 50% scaled feedforward commands failed to converge, so their predictive simulations were performed with the constraints on initial joint angles, joint velocities, and ground reactions removed. To improve computational speed, we also fitted surrogate geometric models of muscle-tendon lengths, velocities, and moment arms using Latin hypercube sampling around joint angle trajectories obtained from one representative walking cycle.

## 3 Results

### 3.1 Neural Control Personalization

**Figure 1.**
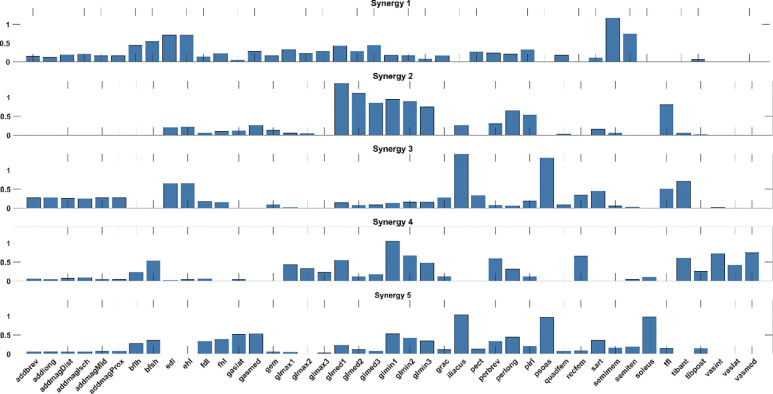
Optimized synergy weights derived from 5 fitting trials.

**Figure 2.**
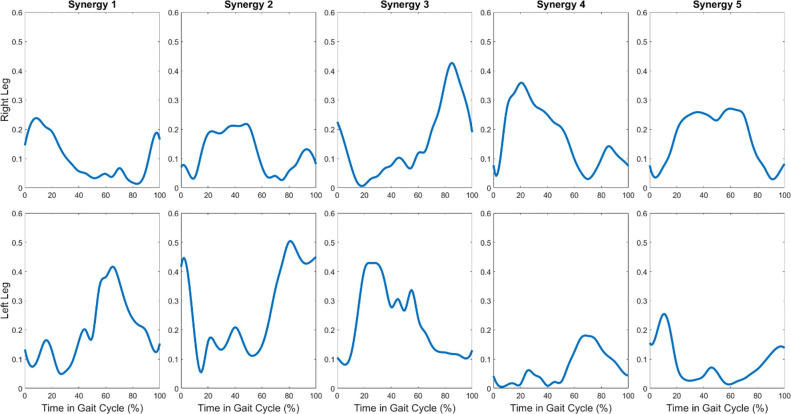
Mean synergy commands from 5 fitting trials used as feedforward commands.

Synergy weights optimized for the 5 fitting trials are shown in Figure 1. The variability accounted for (VAF) of muscle activations within individual trials was 88.46 ± 1.05%. The mean synergy commands for the 5 fitting trials, used as feedforward commands in future steps, are shown in Figure 2.

### 3.2 Feedback Weight Calibration

**Figure 3.**
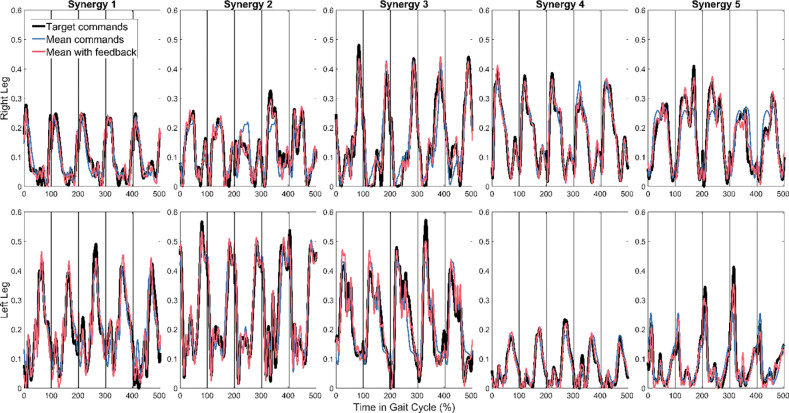
Fit of reference synergy commands (black) by feedback model at 100% feedforward contribution (red) compared to mean synergy commands (blue).

R^2^ correlation coefficients for fitting all 10 synergy commands in all 6 feedforward magnitude cases are shown in Table 1. As an example, the fitting results are plotted for each synergy at 100% feedforward magnitude in Figure 3.

### 3.3 Synergy-Driven Tracking Optimization with Neural Feedback

**Figure 5.**
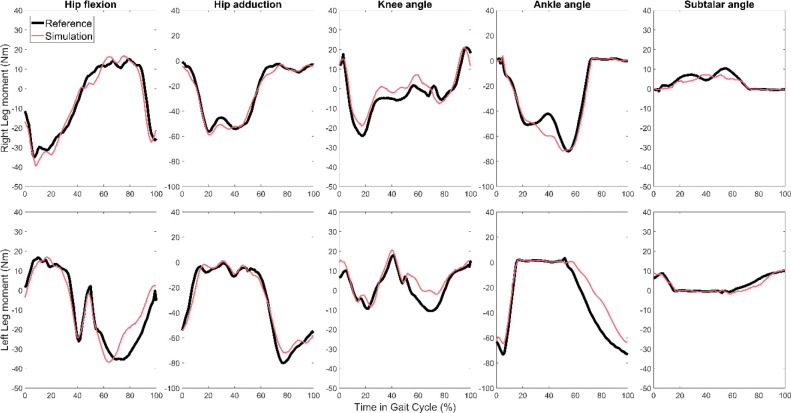
Reference joint moments (black) and predictions using feedback model at 100% feedforward contribution (red) for testing trial 3.

**Figure 6.**
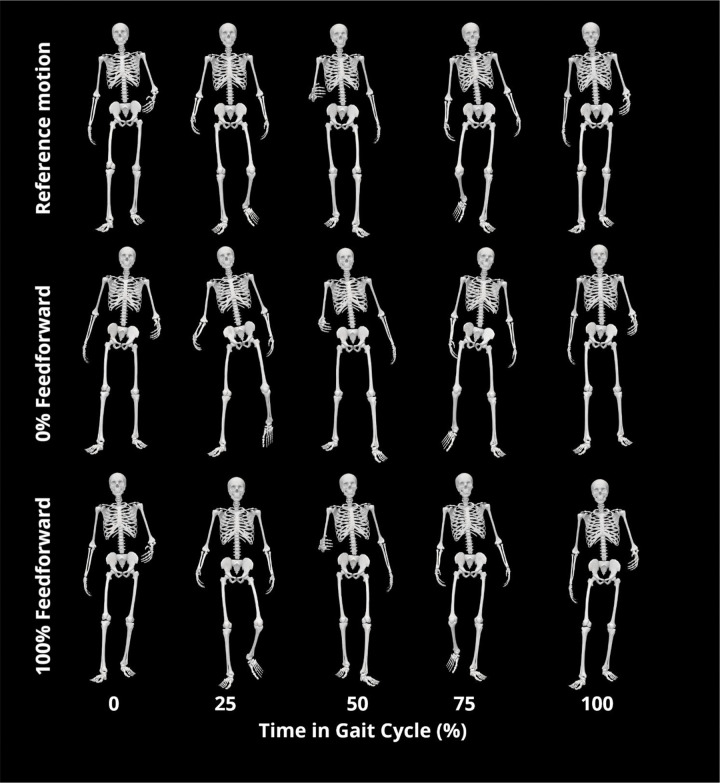
Animation strip of trial 3 reference motion, motion predicted by feedback model at 0% feedforward contribution without initial value constraints, and motion predicted by feedback model at 100% feedforward contribution.

Joint angle, joint moment, and ground reaction errors are shown for all 6 feedforward magnitude cases in Tables 2, 3, and 4, respectively. As an example, joint angle and joint moments are plotted for the most accurate simulation overall, testing trial 3 with 100% feedforward magnitude, in Figure 5. Figure 6 visually compares the nominal dynamically consistent motion of trial 3 with 0% and 100% feedforward magnitude cases.

## 4 Discussion

This study developed a subject-specific neuromusculoskeletal model with a neural feedback model capable of producing walking motions from separate training and testing gait data from a real-life dataset. We fit feedback models and evaluated the models’ predictive abilities for three different testing trials with six levels of averaged feedforward control. The feedback models reproduced synergy commands from fitting trials with high accuracy, achieving R^2^ correlation scores of at least 0.90 in all cases except a single synergy in the 125% feedforward case. The three testing trials had similar levels of accuracy in terms of predicted joint angles, joint moments, and ground reactions, and the most accurate case used a feedforward magnitude of 100%, with feedback making the smallest changes to total synergy commands. The cases using 0%, 25%, and 50% feedforward magnitudes failed to converge while constrained to have similar initial values to nominal data, so we do not claim that these feedback models were able to reproduce any of the 3 testing gait cycles. However, these models were able to produce walking motions that did not satisfy these constraints, though they appeared to represent a significant movement impairment, taking wide, short strides that increased the base of support. This adaptation becomes less pronounced as the feedforward magnitude increases to 100%. These results indicate that while fully feedback-controlled models may walk in simulations (Geyer and Herr, 2010), researchers should be cautious about using such models with real subjects and experimental data. Overall, this study presents a repeatable method for using real data to fit predictive neural feedback models that we find produces dynamically consistent, feasible walking motions.

This study faced multiple technical challenges that needed to be addressed before producing these results that yielded additional notable findings about how to make feedback models work with new data. First, we needed to determine the appropriate number of synergies to use for the neural control structure. We had first used 6 synergies per leg, as that number achieved a higher VAF than the 5 synergy solution, but a neural feedback model with 6 synergies was not as successful as the 5 synergy solution. On closer inspection of the 6 synergy solution, we found that the weights were poorly distributed, including highly sensitive synergies with few muscles. This high sensitivity may be a poor match for a neural feedback model that needs to generalize to predict motions not included in fitting data. Second, again on the note of sensitivity, the feedback weight fitting optimization includes a cost term that penalizes high individual feedback weights, and the weight of this cost term was important to consider. This term was originally added to ensure that solutions would be unique, but it ultimately played a key role in producing functional feedback models. When this term had little emphasis, individual weights could be high, leading to unrealistic situations such as the right knee angle alone holding most of the information on how to form a single synergy. These models failed to generalize. When giving this cost term a higher weight, feedback models could still reproduce fitting synergy command data with high correlation scores, but predictive simulations were also successful. Finally, we noted during the development of the prediction process that including multiple fitting trials is important. As an initial test of our walking simulations with neural feedback, we fitted a neural feedback model at 100% average feedforward magnitude to match a single gait cycle’s synergy commands and tried to predict that single gait cycle. We expected this simulation to be better than any of our predictions of testing cycles as we were fitting and testing the same single gait cycle, and our feedback model’s synergy commands had near-perfect correlation to their target commands. However, this model did not converge to a perfect walking solution, making worse predictions than the 100% feedforward magnitude results presented in this study. This was because the synergy commands the model was trying to fit did not at all time points satisfy a certain one of our constraint types which enforced that joint moments produced by muscles were within 1 Nm of ID moments. The feedback model struggled to adapt to the small changes in control needed, which suggests having a broader selection of fitting data is more important than fitting a small subset of data perfectly, even if only predicting a dynamically consistent motion is a goal, not predicting unseen motions. Future research can assess how much training data feedback models can benefit from to further increase their predictive ability.

Many studies have used feedback control to simulate walking (Geyer and Herr, 2010; Jansen et al., 2014; Falisse et al., 2018; Haeufle et al., 2018), but this study is unique in its modeling fidelity, use of actual data, and predictive ability for testing data. Many feedback models have been two-dimensional (Geyer and Herr, 2010; Haeufle et al., 2018; Aoi et al., 2019, p. 20; Di Russo et al., 2021; Lassmann et al., 2023; Su and Gutierrez-Farewik, 2023; Koseki et al., 2024), which would fail to capture important movement patterns such as increased stance width. Models are often not personalized (Geyer and Herr, 2010; Song and Geyer, 2013; Haeufle et al., 2018; Aoi et al., 2019; Di Russo et al., 2021; Lassmann et al., 2023; Su and Gutierrez-Farewik, 2023, p. 202; Koseki et al., 2024), limiting the accuracy of the model to its subject (Gaudio et al., 2023). Walking motions predicted with feedback control are often only compared to experimental data in broad, population-level terms (Song and Geyer, 2013; Di Russo et al., 2021; Lassmann et al., 2023; Su and Gutierrez-Farewik, 2023; Koseki et al., 2024). Another distancing factor from real data is that experimental EMG data are uncommonly used in fitting the feedback controller (Falisse et al., 2018), while this study uses EMG data to produce muscle activations that are used to fit the neural control structure. This study uses real data and the advanced model personalization capabilities of the NMSM Pipeline to overcome these past limitations while predicting data from outside the feedback fitting process.

Although this study presents significant advancements in modeling neural feedback for a real subject, this work has several limitations that future research will need to address. First, a subset of joint angles is not controlled by the feedback model, most notably the hip rotation and toes, which means the feedback model does not fully control the legs. The methods described in this paper were attempted while including hip rotation in synergy controls and the feedback model, but this version of the process yielded unrealistic joint angles, including high knee extension, issues which were largely not present when the coordinate was removed. This could be a limitation of the hip rotator muscles in the musculoskeletal model used, such as weaker muscles than required or inaccurate muscle paths, or a limitation of the motion capture of the hip rotation coordinate. Second, it is important to note that no muscle-specific feedback inputs were included, even though muscle-specific feedback is likely important for feedback control given its basis in quantities such as the stretch reflex and Golgi tendon organs (Geyer and Herr, 2010; Jansen et al., 2014; Trompetto et al., 2014; Ang et al., 2018; Falisse et al., 2018; Haeufle et al., 2018). Accurately modeling muscle-specific feedback becomes more complex in detailed three-dimensional models as relationships between selected muscle pairs such as agonist-antagonist interactions are not always well-defined and can vary depending on the state of the model or the coordinate of interest. Identifying how to include these interactions in a feedback model with detailed three-dimensional geometry is significant and important work. Third, our feedback model produced synergy commands, and we did not test a feedback model that produced individual muscle activations. Past work indicates that feedback models perform well working at the synergy command level (Safavynia and Ting, 2013; Aoi et al., 2019), but feedback to individual muscles may have given the model finer control and improved its predictive ability. Fourth, our muscle models have rigid tendons and do not account for short-range stiffness. Rigid tendons are likely sufficient for walking (Millard et al., 2013), but short-range stiffness is important for even slight movements (De Groote et al., 2017). Fifth, our feedback model is instantaneous, not including a time delay between feedback input and output quantities. This delay is a known component of feedback models (Geyer and Herr, 2010), though similar past work with complex neuromusculoskeletal models has not included it (Jansen et al., 2014). Ground reactions were also not included in our feedback model inputs. They are important to the timing of feedback mechanisms (Geyer and Herr, 2010), but their high sensitivity to small kinematic changes made them difficult to include as a feedback model input during optimal control simulations, so joint moments were included as a surrogate quantity for ground reactions.

In conclusion, we have shared a novel method of producing gait simulations with neural feedback control based on real data with the ability to combine feedback control with varying levels of feedforward commands. This method incorporates dense, subject-specific data for highly personalized models and solutions, bringing new possibilities to OpenSim models familiar to researchers. With further work along these lines with additional data, accurate and flexible feedback models may promote the development of new techniques for studying movement impairments and patient-specific treatment design.

## Figures and Tables

**Figure 4.**
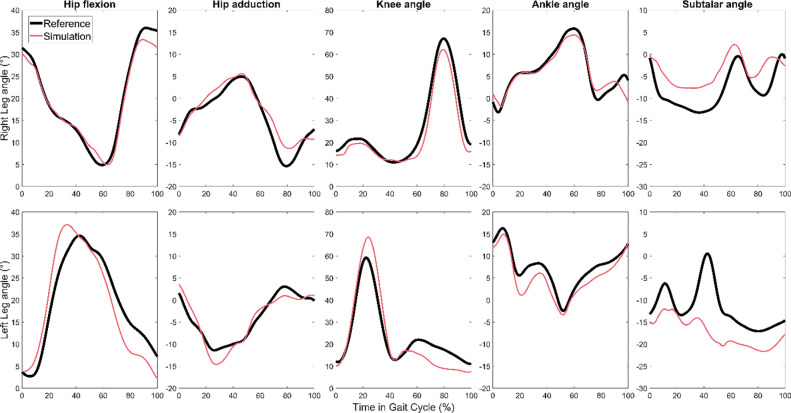
Reference joint angles (black) and predictions using feedback model at 100% feedforward contribution (red) for testing trial 3, the most successful trial overall.

**Table 1.** R^2^ correlation coefficients for fitting synergy commands at each FF contribution level.

FF contribution	0%	25%	50%	75%	100%	125%
Synergy 1 (R)	0.978	0.976	0.973	0.959	0.950	0.939
Synergy 2 (R)	0.981	0.978	0.973	0.943	0.928	0.908
Synergy 3 (R)	0.975	0.976	0.975	0.967	0.963	0.957
Synergy 4 (R)	0.981	0.986	0.987	0.982	0.979	0.971
Synergy 5 (R)	0.977	0.979	0.980	0.958	0.953	0.946
Synergy 1 (L)	0.960	0.959	0.954	0.931	0.917	0.898
Synergy 2 (L)	0.992	0.991	0.989	0.977	0.967	0.953
Synergy 3 (L)	0.947	0.945	0.941	0.927	0.915	0.900
Synergy 4 (L)	0.981	0.981	0.979	0.961	0.948	0.930
Synergy 5 (L)	0.959	0.960	0.959	0.931	0.919	0.903

**Table 2.** Root mean square error (RMSE) (mean ± standard deviation) in predictions of joint angles included on right (R) and left (L) sides as feedback model inputs for each FF contribution level and all 3 testing trials.

FF contribution	0%	25%	50%	75%	100%	125%
Hip flex. (R)	16.3 ± 0.93	12.8 ± 0.72	8.45 ± 1.27	7.06 ± 0.79	2.89 ± 1.71	2.41 ± 1.15
Hip add. (R)	12.3 ± 3.01	8.83 ± 1.49	4.93 ± 0.40	1.84 ± 0.50	2.01 ± 0.09	2.55 ± 0.41
Knee angle (R)	19.7 ± 3.00	17.9 ± 0.21	15.3 ± 0.58	11.0 ± 0.90	4.87 ± 0.67	6.49 ± 0.60
Ankle angle (R)	12.5 ± 0.50	8.60 ± 0.90	9.56 ± 1.08	3.52 ± 0.26	1.45 ± 0.20	2.93 ± 0.95
Subtalar (R)	14.5 ± 1.53	5.35 ± 0.68	12.3 ± 2.10	4.05 ± 0.64	5.29 ± 0.43	3.61 ± 0.29
Hip flex. (L)	15.7 ± 1.80	9.98 ± 0.88	6.44 ± 0.93	4.36 ± 1.59	4.36 ± 0.71	6.10 ± 0.57
Hip add. (L)	13.4 ± 2.76	9.85 ± 1.27	5.86 ± 0.40	2.87 ± 0.71	2.51 ± 0.99	4.73 ± 1.09
Knee angle (L)	15.4 ± 1.02	13.3 ± 1.11	12.6 ± 1.71	7.66 ± 1.20	6.03 ± 0.37	9.25 ± 1.25
Ankle angle (L)	9.71 ± 1.36	7.54 ± 1.19	10.9 ± 1.11	4.48± 0.84	2.12 ± 0.44	2.64 ± 0.08
Subtalar (L)	16.7 ± 3.43	8.38 ± 0.87	21.7 ± 2.29	8.86 ± 1.23	7.72 ± 0.78	5.87 ± 1.74

**Table 3.** RMSE (mean ± standard deviation) in predictions of joint moments included as feedback model inputs for each FF contribution level and all 3 testing trials.

FF contribution	0%	25%	50%	75%	100%	125%
Hip flex. (R)	15.9 ± 2.35	11.1 ± 2.17	6.17 ± 1.69	4.23 ± 0.73	5.35 ± 1.58	6.85 ± 1.38
Hip add. (R)	33.7 ± 0.88	25.6 ± 0.77	14.8 ± 1.04	7.19 ± 0.60	3.41 ± 0.40	8.43 ± 1.01
Knee angle (R)	13.3 ± 2.53	15.4 ± 2.67	16.9 ± 3.29	12.9 ± 1.85	5.61 ± 2.10	8.42 ± 1.59
Ankle angle (R)	27.9 ± 3.10	21.5 ± 2.25	15.5 ± 2.34	8.41 ± 2.17	9.00 ± 3.01	21.2 ± 2.82
Subtalar (R)	2.82 ± 0.97	1.44 ± 0.79	3.03 ± 1.02	1.67 ± 0.47	1.43 ± 0.46	2.74 ± 0.25
Hip flex. (L)	20.2 ± 1.23	15.6 ± 1.46	10.6 ± 1.09	6.06 ± 1.96	5.85 ± 2.47	8.02 ± 3.23
Hip add. (L)	41.6 ± 1.46	34.9 ± 0.76	23.6 ± 1.27	11.9 ± 0.52	5.98 ± 1.05	8.68 ± 2.68
Knee angle (L)	8.26 ± 1.52	8.53 ± 2.18	9.37 ± 2.54	7.79 ± 2.58	7.49 ± 2.38	7.64 ± 1.59
Ankle angle (L)	26.4 ± 7.16	23.8 ± 6.40	18.6 ± 6.79	11.4 ± 6.95	9.64 ± 1.96	11.8 ± 1.29
Subtalar (L)	3.24 ± 0.84	3.17 ± 0.83	4.38 ± 0.41	2.16 ± 0.34	1.76 ± 0.44	1.90 ± 0.18

**Table 4.** RMSE (mean ± standard deviation) in predictions of ground reactions (anterior force, vertical force, lateral force, frontal moment, transverse moment, and sagittal moment) for each FF contribution level and all 3 testing trials.

FF contribution	0%	25%	50%	75%	100%	125%
Ant. F (R)	38.6 ± 2.23	29.0 ± 4.74	19.9 ± 3.72	13.3 ± 1.93	13.5 ± 2.84	22.4 ± 6.75
Vert. F (R)	172 ± 35.3	128 ± 11.3	63.4 ± 9.94	58.4 ± 3.10	59.5 ± 24.5	70.2 ± 20.5
Lat. F (R)	27.6 ± 2.30	21.5 ± 3.85	15.0 ± 4.87	11.2 ± 4.79	9.71 ± 2.31	9.39 ± 1.82
Front. M (R)	5.17 ± 0.38	2.62 ± 0.59	2.65 ± 1.10	1.83 ± 0.32	1.54 ± 0.46	2.77 ± 0.32
Trans. M (R)	4.54 ± 0.28	3.17 ± 0.39	2.00 ± 0.17	1.85 ± 0.21	1.89 ± 0.20	3.36 ± 0.54
Sag. M (R)	26.4 ± 2.46	23.0 ± 1.99	16.0 ± 2.70	9.15 ± 2.07	7.54 ± 2.99	20.6 ± 3.00
Ant. F (L)	44.2 ± 1.41	29.4 ± 2.86	12.7 ± 3.26	16.1 ± 3.62	25.7 ± 6.30	23.1 ± 4.88
Vert. F (L)	165 ± 18.5	115 ± 26.0	67.1 ± 10.5	62.1 ± 8.08	60.9 ± 13.2	70.2 ± 17.8
Lat. F (L)	31.9 ± 5.81	23.4 ± 0.42	19.8 ± 1.06	19.1 ± 0.45	11.8 ± 0.97	11.0 ± 2.92
Front. M (L)	5.45 ± 1.26	3.83 ± 0.57	3.69 ± 0.64	2.18 ± 1.05	2.41 ± 0.26	2.34 ± 0.44
Trans. M (L)	4.31 ± 0.25	2.07 ± 0.36	1.76 ± 0.29	2.94 ± 0.84	1.18 ± 0.20	2.31 ± 0.42
Sag. M (L)	28.9 ± 8.53	26.5 ± 6.13	15.3 ± 5.99	11.6 ± 6.16	9.50 ± 1.79	11.6 ± 1.80
